# Bombesin Receptor Family Activation and CNS/Neural Tumors: Review of Evidence Supporting Possible Role for Novel Targeted Therapy

**DOI:** 10.3389/fendo.2021.728088

**Published:** 2021-09-01

**Authors:** Terry W. Moody, Lingaku Lee, Irene Ramos-Alvarez, Tatiana Iordanskaia, Samuel A. Mantey, Robert T. Jensen

**Affiliations:** ^1^Department of Health and Human Services, National Cancer Institute, Center for Cancer Training, Office of the Director, Bethesda, MD, United States; ^2^Digestive Diseases Branch, National Institute of Diabetes and Digestive and Kidney Diseases, National Institutes of Health, Bethesda, MD, United States; ^3^Department of Gastroenterology, National Hospital Organization Kyushu Cancer Center, Fukuoka, Japan

**Keywords:** bombesin, gastrin releasing peptide, neuromedin B, glioma, neuroblastoma, medulloblastoma, gastrin-releasing peptide, central nervous system tumor

## Abstract

G-protein-coupled receptors (GPCRs) are increasingly being considered as possible therapeutic targets in cancers. Activation of GPCR on tumors can have prominent growth effects, and GPCRs are frequently over-/ectopically expressed on tumors and thus can be used for targeted therapy. CNS/neural tumors are receiving increasing attention using this approach. Gliomas are the most frequent primary malignant brain/CNS tumor with glioblastoma having a 10-year survival <1%; neuroblastomas are the most common extracranial solid tumor in children with long-term survival<40%, and medulloblastomas are less common, but one subgroup has a 5-year survival <60%. Thus, there is an increased need for more effective treatments of these tumors. The Bombesin-receptor family (BnRs) is one of the GPCRs that are most frequently over/ectopically expressed by common tumors and is receiving particular attention as a possible therapeutic target in several tumors, particularly in prostate, breast, and lung cancer. We review in this paper evidence suggesting why a similar approach in some CNS/neural tumors (gliomas, neuroblastomas, medulloblastomas) should also be considered.

## Introduction

G-protein-coupled receptors (GPCRs) are increasingly being considered as possible therapeutic targets in cancer (1–3). This is occurring not only because activation of GPCR on tumors can have potent tumor growth effects, but also because they are frequently over-/ectopically expressed on tumors and thus can be used for targeted therapy (1–4). GPCR expression by tumors is a frequent finding, not only in the percentage of a given tumor type showing over-/ectopic expression, but also in that many different tumors demonstrate this phenomenon, and furthermore, in the numerous different classes of GPCRs that can be over-/ectopically expressed (2, 4–7). One of the GPCR classes that is receiving particular interest is the human bombesin receptor (BnR) family (8–12). This is occurring because the Bn family of receptors is one of the most frequently overexpressed GPCRs in a number of common, malignant tumors (breast, prostate, lung, pancreas, head/neck squamous cell tumors, colon, CNS) (8–10, 13–19). Of this group, the possible therapeutic utility of BnR over-/ectopic expression in prostatic cancer (4, 20–22) and breast cancer (4, 23–25) is receiving the most attention, whereas its possible therapeutic utility in a number of these other cancers has received much less attention. In this review we concentrate on the roles of BnRs in various CNS/neural tumors, particularly concentrating on gliomas, neuroblastomas, and medulloblastomas. Gliomas are the most frequent primary malignant brain/CNS tumor (26, 27) with glioblastoma having a 10-year survival of <1%; neuroblastomas are the most common extracranial solid tumor in children with (28) with long-term survival <40%, and medulloblastomas are less common, but one subgroup has a 5-year survival <60% (29). Therefore, there is an increasing need for new therapeutic approaches in these tumors; hence, the possible evidence for considering BnR-linked strategies is reviewed here.

Prior to reviewing the BnRs’ role in each of these tumors, a short introduction into the BnR family of receptors will follow to allow the reader to make a better assessment of the subsequent data.

## Bn/BnR Family of Receptors

### Bn/BnR: General

The human Bn receptor family includes three different GPCRs [i.e., the Neuromedin B receptor (NMBR) (BB_1_), the Gastrin-releasing peptide receptor (GRPR) (BB_2_), and the orphan GPCR, bombesin-receptor subtype 3(BRS-3) (BB_3_)] (12, 14, 30, 31). Because of their widespread, common use in many clinical papers, these will be referred to as NMBR, GRPR, and BRS-3 in this paper, instead of the official NC-IUPHAR nomenclature of BB_1_, BB_2_, and BB_3_, which is less familiar to readers in this area (30, 31). This general class of peptide receptors is referred to as BnRs, because the original member of the class of peptides interacting with these receptors was called Bombesin, which is a tetra-decapeptide isolated from the skin of the European frog *Bombina bambina* (12, 32, 33). Subsequently, a large number of related peptides were isolated from different frog skins (12, 34, 35), as well as the mammalian-equivalent peptide, GRP, a 27-amino acid peptide that shares a close identity to Bn (36), as well the mammalian decapeptide, NMB, which shares identities to the frog peptide ranatensin, which was isolated from the North American frog *Rana pipiens* (12, 34, 37). In mammals including humans, GRPR is endogenously activated by GRP/GRP(10-27) and the NMBR by NMB (12, 14, 30, 31). The human BRS-3 (hBRS-3) is included in the BnR group because of its high homology to the hGRPR (51% amino acid identities) and hNMBR (47% amino acid identities) (12, 31, 38); however, at present it remains an orphan receptor because its natural ligand is unknown.

### Bn/BnR: Pharmacology

The biological activity of both NMB and GRP is primarily determined by the amino acids in the carboxyl terminus, which is amidated, with GRP having the same last seven carboxyl-terminal amino acids as Bn (i.e., Trp-Ala-Val-Gly-His-Leu-Met-NH_2_), but differing from NMB, which varies from GRP in two identities in its last seven carboxyl amino acids (underlined) (i.e., Trp-Ala-Thr-Gly-His-Phe-Met-NH_2_) (12, 14, 36, 37). GRP is highly selective for the GRPR over the NMBR (740-fold), as is NMB for the NMBR over the GRPR (700-fold), whereas Bn is relatively unselective (20-fold GRPR over NMBR) (14, 39–41). Numerous Bn-related/peptide analogs (13, 14, 20, 39, 42–44) as well as a few non-peptide/peptoid compounds (14, 45, 46) have been described, which are selective GRPR or NMBR agonists. Furthermore, there are numerous classes of selective GRPR and NMBR antagonists (12, 14, 20, 47–59). Similarly, recently both selective agonists (60–68) and antagonists (69–72) have been described for the BRS-3 receptor. Both selective agonists and antagonists or related compounds are increasingly being used to determine the roles of BnRs in various physiological and pathologic processes including effects on tumor biology. Furthermore, they are increasingly being used to target cytotoxic agents to tumors (4, 10, 13, 20, 73, 74). Frequently different tumors express different BnRs, and therefore, a universal ligand interacting with all BnRs with high affinity would be of value in human studies of imaging or targeted cytotoxicity. One such ligand, [D-Phe^6^,β-Ala^11^,Phe^13^, Nle^14^]bombesin-(6-14), has been described (39, 75–77), and this Bn analogue (or related Bn analogues) is being increasingly investigated for such therapeutic approaches (78–81).

### Bn/BnR: Distribution

GRP and NMB, as well as their receptors, are widely distributed in the CNS and peripheral tissues (12, 14, 30, 68, 82–84). In the monkey CNS, both GRPR and NMBR mRNA were found in the hypothalamus, thalamus, amygdala, cerebellum, hippocampus, caudate nucleus, and spinal cord (83). In binding studies in rat CNS, a high density of NMBR was found in the olfactory regions and central thalamic nuclei, with the highest density of GRPRs in the hypothalamus, especially in suprachiasmatic and paraventricular nuclei, which is in agreement with the mRNA distribution of these two receptors in rat brain (85). In the monkey brain, BRS-3 mRNA is found throughout the CNS, with the highest amounts in the hypothalamus, whereas BRS-3 mRNA is found in low amounts in most peripheral tissues, with the highest found in the testis and followed by the pancreas, ovary, thyroid, and pituitary gland (83).

Activation of the BnRs in many tumors has a potent effect on their biological behavior (increase growth, migration, invasion, development of metastases), often in an autocrine manner (8–10, 12, 86–88). Because of the presence and potent growth effects of BnR activation on tumor behavior, recently, there is increased interest in utilizing BnRs for novel therapeutic approaches (10–12, 89–91).

### Bn/BnR: Cellular Signaling

The three BnR subtypes couple primarily to the Gq/11 and G12/13 family of G proteins (12, 14). The main signaling cascade by all BnRs is the activation of the phospholipase C (PLC) cascade with stimulation of changes in cytosolic [Ca^2+^]_i_, the generation of diacylglycerol, and the activation of protein kinases Cs (PKCs) (12, 14, 19, 92–95). Also, a larger number of other cellular signaling cascades are activated. These include the activation of other phospholipases (A, D); protein kinase D activation; MAP kinases (ERK1/2, JNK, p38); PI3K/Akt; Src kinases; activation of numerous ion channels and the mTor pathway (12, 14, 31, 93).

Recently, the ability of each of the three BnRs to activate two novel signaling cascades is receiving increased attention. This has occurred due to the increased recognition that, similar to numerous growth-factor receptors, BnR activation can also activate numerous tyrosine kinase cascades (p125^FAK^, PYK-2, paxillin, p130^CAS^, etc.) (9, 12, 14, 19, 40, 47, 71, 96–100). Secondly, it is being increasingly recognized that mediation of many of the growth-related effects of BnRs, especially in neoplastic tissues, are due to the transactivation of the epidermal growth factor receptor family (EGFR, HER-2, HER-3, HER-4) (101–103). BnR-mediated transactivation of EGFR/HER2Neu family of receptors (EGFR, HER-2, HER-3) has been shown to involve activation of PLC with mobilization of cellular calcium and PKC activation, Src kinase activation, stimulation of matrix metalloproteinases, and shedding of EGF-related ligands, as well as the stimulation of reactive oxygen species (102, 104–107).

Post receptor activation, each of the Bn receptor subtypes undergo a number of processes, similar to other GPCRs, including receptor phosphorylation, receptor internalization, downregulation, and desensitization (12, 47, 93, 97, 98, 108–113).

### Bn/BnR: Physiological/Pathophysiological Effects

Activation of GRPR and NMBR results in a wide range of physiological/pathophysiological actions, including the stimulation of smooth-muscle contraction (particularly urogenital and gastrointestinal tract), gastrointestinal motility, as a modulator of immune function (stimulate phagocytosis, chemo-attraction of monocytes, macrophages, neutrophils; stimulate macrophage IL-1 release); secretion [pancreatic, gastric, intestinal, endocrine (insulin)], hormone secretion (release of LH, GnRH, prolactin, growth hormone, TSH, CCK, GLP1, enteroglucagon, GIP, PP, neurotensin), and stimulation of a wide range of CNS effects (feeding, behavioral effects, body temperature control, regulation of circadian rhythm, sighing) and functioning as an important spinal neurotransmitter mediating pruritus (12, 106, 114–120). A physiological role for the BRS-3 receptor has yet to be completely defined; however, recently studies suggest an important role in metabolic homeostasis, in glucose and insulin regulation, in obesity and diabetes mellitus, in the regulation of body temperature, and in feeding behavior (14, 44, 68, 121–123). For each of the three BnR subtypes, numerous studies support them playing important roles in regulating the growth of normal/neoplastic tissues (3, 9, 10, 12, 14, 21, 42, 102, 124, 125).

## Presence and Effects of Bombesin-Related Peptides on CNS/Neural Tumors

### General

Numerous studies have reported in CNS and peripheral neural tumors the presence and the tumoral effects of both mammalian bombesin (Bn)-related peptides [gastrin-releasing peptide [GRP], neuromedin B (NMB)] and their receptors [GRPR (BB2), NMBR (BB1)], as well as the mammalian BnR family orphan receptor [bombesin receptor subtype 3 (BRS-3) (BB3)]. These studies include the effects of BnR activation on tumoral signaling, growth, and other behaviors. Also, as discussed above in non-CNS/neural tumors, in CNS/neural tumors, there is increased interest recently in using the presence of BnRs in these tumors for both tumor localization and possible therapeutic approaches, as well as strategies directed at inhibiting the growth-promoting effects of BnRs in these tumors. Below, each of these areas will be briefly reviewed, emphasizing results from the most recent studies.

BnR’s effects on CNS/neural tumors in most studies involve one of three tumoral types—gliomas or neuroblastomas or medulloblastomas—and these will be each reviewed below.

### Bn-Related Peptides and Gliomas

#### Gliomas: General

Gliomas account for 26.5% of all primary brain/CNS tumors, comprising 80.7% of all the malignant brain/CNS tumors, making them the most frequent, primary malignant brain/CNS tumor (26, 27). Gliomas have an annual adjusted incidence rate of 6.0 per 100,000 population and are considered to arise from glial cells (astrocytes, oligodendrocytes, microglia, ependymal cells) or stem cells that have glial cell properties on transformation (126, 127). Glioblastomas account for the majority of gliomas (56.1%) and have a 5-year survival rate of only 5%, with a 10-year survival rate of 0.76% (26, 27, 127, 128).

Gliomas are heterogenous and are classified based on their pattern of growth (diffuse *vs* non-diffuse), on microscopic similarities to their putative cell of origin, on tumor grade (I–IV), and on various molecular features (126, 129). Molecular features that are particularly important are the presence or absence of isocitric dehydrogenase type 1/2 (IDH) (present in the majority of WHO Grade I and II diffuse astrocytic/oligodendroglia tumors), the presence of 1p/19q codeletions (a defining feature of oligodendroglial tumors), and the presence of O6-methyl guanine methyl-transferase methylation(MGMT) mutation/inactivation] (126, 129). The presence of these molecular markers is of particular importance because they identify subgroups with improved prognosis and, in the case of MGMT inactivation, improved response to TMZ treatment (126, 129).

Treatment involves primarily surgery, radiation, and chemotherapy, but for the glioblastomas, which are the majority, despite improvements in prognostic markers, there has been little improvement in survival (27, 130–132). Therefore, particularly in the case of glioblastomas, there is increased interest in newer approaches in treatment. In other non-CNS tumors (as reviewed earlier) the ectopic expression/overexpression of various peptide receptors is increasingly being applied and investigated for its possible diagnostic and therapeutic approaches for treatment of these tumors (10, 73, 124, 125, 132–136). In the case of gliomas and particularly for glioblastomas, the over-/ectopic expression of the BnR family has been investigated in a number of studies and may provide new therapeutic approaches, as recently used in non-CNS tumors (prostate, colon, etc.) (10, 13, 20, 22, 132, 137) and will be reviewed in the following section.

#### Gliomas: Bn/BnR Expression

In an immunocytochemical study of 34 cases of human gliomas [WHO grade 1 astrocytomas (three cases), grade 2 (four cases), grade 3 (three cases), and grade 4-glioblastoma multiforme (24 cases)], GRPR was detected in 100% (138). GRPRs were detected in both the tumoral tissue in all patients as well as in tumor-associated endothelial cells, but in the normal brain tissue, GRPRs were only detected in the neurons and not in glial cells (138). In a second study (139), Bn immunoreactivity was detected in each of the six gliomas studied, in two out of three pilocytic astrocytomas, and in three of four ependymomas studied. In contrast, no GRPRs were detected in normal glial cells, but were found in brain neurons (138). In one study (140) involving 46 different gliomas, Bn immunoreactivity content in all was below the level of detection, whereas in a CNS metastatic oat cell of the bronchus, the Bn immunoreactivity level was markedly elevated.

**Figure 1:**
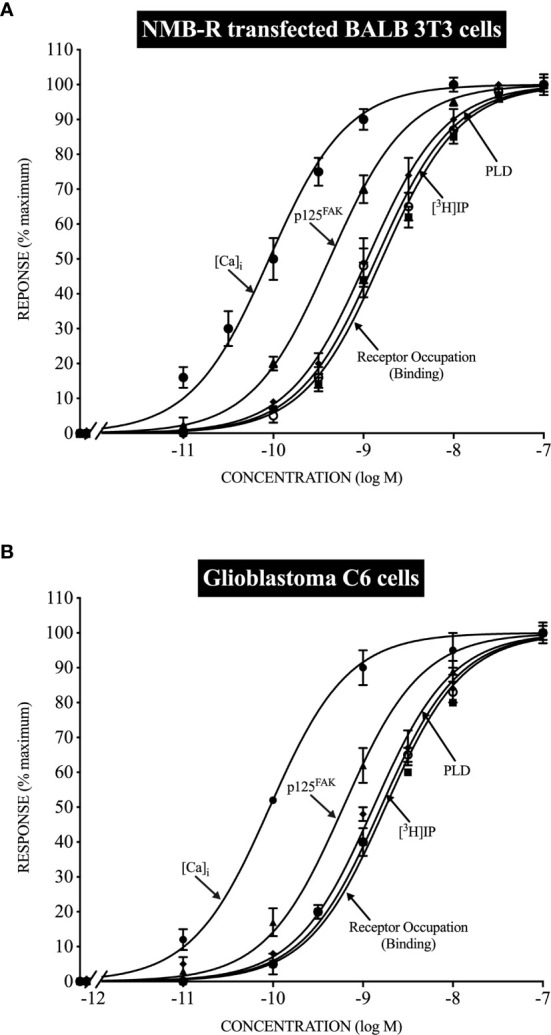
Relationship between the ability of Neuromedin B to occupy NMB receptors (NMBR) and stimulate various intracellular signaling cascades in normal NMBR transfected cells (top, panel **A**) and Glioblastoma C6-cells (bottom, panel **B**). Stoichiometric relationships are drawn from data in (150, 151) and show the almost identical coupling in normal NMBR transfected cells (top) and Glioblastoma C6-cells (bottom) for NMBR occupation to activate phospholipase C, resulting in increases in cytosolic calcium ([Ca)_i_] and stimulate the generation of phosphoinositides (^3^H]IP); to activate phospholipase D, and the activation of p125focal adhesion kinase (p125^FAK^).

Expression, as well as overexpression, of BnRs has been reported in both human (138, 139, 141–145) and rat (142, 146) gliomas/glioma cell lines (147, 148). In one study (143), 85% of the adult human glioblastoma cell lines (U-373MG, D-247MG, U-118MG, U-251MG, D-245MG, U-105MG, D-54MG, A-172MG, D-373MG) and pediatric glioblastoma cell lines (SJ-S6, SJ-G2) studied were reported to possess functional (increasing cytosolic [Ca^2+^]_i_) BnRs, and pharmacological studies demonstrated they were of the GRPR subtype. In another study of six human glioblastoma cell lines (U-138, U-118, U-1242, U-87,U-372, U-563), all expressed GRPR receptors detectable by binding studies; however, in none was immunoreactive BN/GRP-related peptide detected (<0.1 pmol mg protein on radioimmunoassay) (141). In this study (141), the density of the GRPR receptors in the different glioblastoma cell lines varied markedly, with U-118/U-138 possessing greater than 20-fold more than U-572/U-563 glioblastoma cell lines (141). In the human glioblastoma cell lines U138-MG, U-87, and U-373, which have high levels of GRPR receptors and are responsive to GRPR activation, GRPRs’ presence has been detected by both GRPR mRNA expression and immunohistochemical studies (8, 144, 149). In one study GRPRs were identified in rat glioma cells by both PCR and immunocytochemically, whereas in a number of other studies, in contrast to findings in human gliomas where GRPRs are reported, in rat glioblastoma C-6 studies, by binding studies and functional studies, NMBR receptors were found (146–148) (**Figure 1**). In another study (152) the NMBR in C-6 rat glioma cells was further characterized and compared to the human NMBR. Both NMBR receptors (152) were found to be of similar molecular weight on receptor cross-linking studies (Mr-63,000), to have similar NMB affinity (4–6 nM), and the NMBR in both cells to be an N-linked sialoglycoprotein, not containing disulfide bonds or O-linked carbohydrates, but to have two tri-antennary and/or tetra-antennary complex oligosaccharide bonds.

One study (153) investigated the mRNA expression levels of each of the human BnRs (GRPR, NMBR, BRS-3) in recurrent gliomas in nine patients. This is the only study to date that has included any data on BRS-3 in these tumors. All three BnR subtypes mRNA were found in all tumors, with the highest levels for BRS-3 in all tumors, followed by GRPR, and the lowest for NMBR (153). The relative expression levels of the different BnRs in the different gliomas did not correlate with each other and thus were independently expressed for each of the three genes (153).

#### Gliomas: Bn/BnR Signaling

A number of studies on adult human glioblastoma cell lines (141, 143), as well on rat glioblastoma cell lines (C-6) (147, 150, 154–156), report Bn-related peptides stimulate tumor cell activation, assessed by measuring changes in cytosolic calcium (96, 141, 143, 147, 150, 151, 154–158) and stimulating the generation of phosphoinositides (96, 147, 150, 156, 157), effects supporting the role of activation of phospholipase C as an important signaling cascade in these tumors, similar to shown in studies of BnR activation in other tissues (14, 47, 93, 150). In studies on various human glioblastoma cell lines, evidence was provided that the activation of phospholipase C by Bn-related peptides, as well as MAP kinase activation, was due to the activation of GRPRs (GRPR selective antagonism) with GRP more potent than NMB (143). In contrast, in the rat glioblastoma cell line C-6, the activation of phospholipase C, resulting in the mobilization of cellular calcium as well as generation of phosphoinositides, was due to activation of NMBRs (demonstrated by selective agonist and antagonists) (147, 150, 151, 154) (**Figure 1**). In rat C-6 glioma cells (147, 150, 151, 154, 157), the activation of the NMBR, in addition to increasing cytosolic calcium levels, stimulated the generation of arachidonic acid release, increased tyrosine phosphorylation of p125 focal adhesion kinase [p125^FAK^], activation of phospholipase D, and transiently elevated c-fos expression. A comparison of the stoichiometry (96, 150, 151) of NMBR occupation, mobilization of cellular calcium, generation of phosphoinositides, activation of phospholipase D, and stimulation of p125 FAK tyrosine phosphorylation, between NMBR activation on rat C-6 glioma cells and human NMBRs in a normal cell, showed results that were superimposable, and furthermore, the degree of receptor spareness in NMBR occupation and activation of the different signaling cascades was similar, demonstrating the NMBR on these tumor cells and NMBR in human cells had identical signaling coupling (**Figure 1**). Other studies demonstrated that the NMBR on rat glioma C-6 cells underwent desensitization, downregulation with internalization, and desensitization in a similar manner to human NMBRs transfected in normal cells (96, 148, 154). Rat C-6 glioma cells were found not to possess voltage-operated Ca^2+^ channels (158), and thus, it was proposed as that they could be a good model to investigate receptor-operated Ca^2+^ channels that are also seen on native glial cells (158). In human glioblastoma U-87-MG cells, as well as U-373MG cells, GRP (14–27) induced the expression of c-fos and c-jun mRNA, and this increase was prevented by treatment with a GRPR antagonist. Furthermore, treatment with the GRPR antagonist of nude mice with xenografts of U-87MG decreased the tumor size by 60%, also decreased the c-fos levels by 30–40%, leading the authors to suggest that downregulation of the c-fos oncogene by the GRP antagonism could be one of the mechanisms for its antigrowth effects.

#### Gliomas: Bn/BnR Affect Tumor Growth/Proliferation

BnR antagonists inhibited the growth of xenografts of human glioblastomas cell lines (U-87G, U-373MG) in nude mice (138, 145, 159, 160) in addition to inhibiting the growth of rat glioblastoma cell line C-6, when present alone (149, 154, 161), as well as increased the effectiveness of growth inhibition by temozolomide (142, 161). *In vivo* studies demonstrate that GRPR antagonists (RC-3095) inhibited the growth of the C-6 glioma by 60%, and when combined with temozolomide, a further reduction in tumor size occurred (161). In one study of human glioblastoma U-87MG cells (160), both GRPR and NMBR mRNA was found; however, GRP mRNA was not found, leading the authors to speculate that the GRP effect on these tumor cells was likely mediated by a paracrine mechanism, although no additional studies were performed to establish what cells might be secreting the GRP. In human A172 glioblastoma cells (162), knockdown of GRPR resulted in the development of cell senescence, which was accompanied by increases in p53, p21, and p16, as well as activation of EGF receptors and a reduction of p38.

Stimulation of BnRs in various human glioblastoma cell lines (U-373MG, D-247MG) resulted in activation of the MAP kinase cascade (143), as well as increases in DNA synthesis (143). In the human glioblastoma cell line U-373MG, Bn peptides (GRP, NMB) stimulate DNA synthesis, which is mediated by GRPR (163). In the human glioblastoma cell line U-138-MG, GRP stimulated proliferation when combined with agents that increase cellular cAMP (forskolin, 8-Br-cAMP, PDE inhibitor), but not when either was present alone (144). The GRPR antagonists RC-3095, RC-3049-III, RC-3049-Et inhibited the proliferation of the human glioblastoma cell lines U-87-MG, U-373-MG, and U-118MG (138, 149) and in U-118MG (145); the tumoral expression of VEGF; the expression of PKC-alpha; and the Bcl-2:Bax ratio, indicating a net apoptotic gain. In an experimental study (164) using intracranial administration of the mouse glioblastoma cell line, CT-2A to make CNS tumors, the administration of the small molecule 77427, which is a GRPR antagonist, inhibited angiogenesis, and smaller tumors developed.

**Figure 2:**
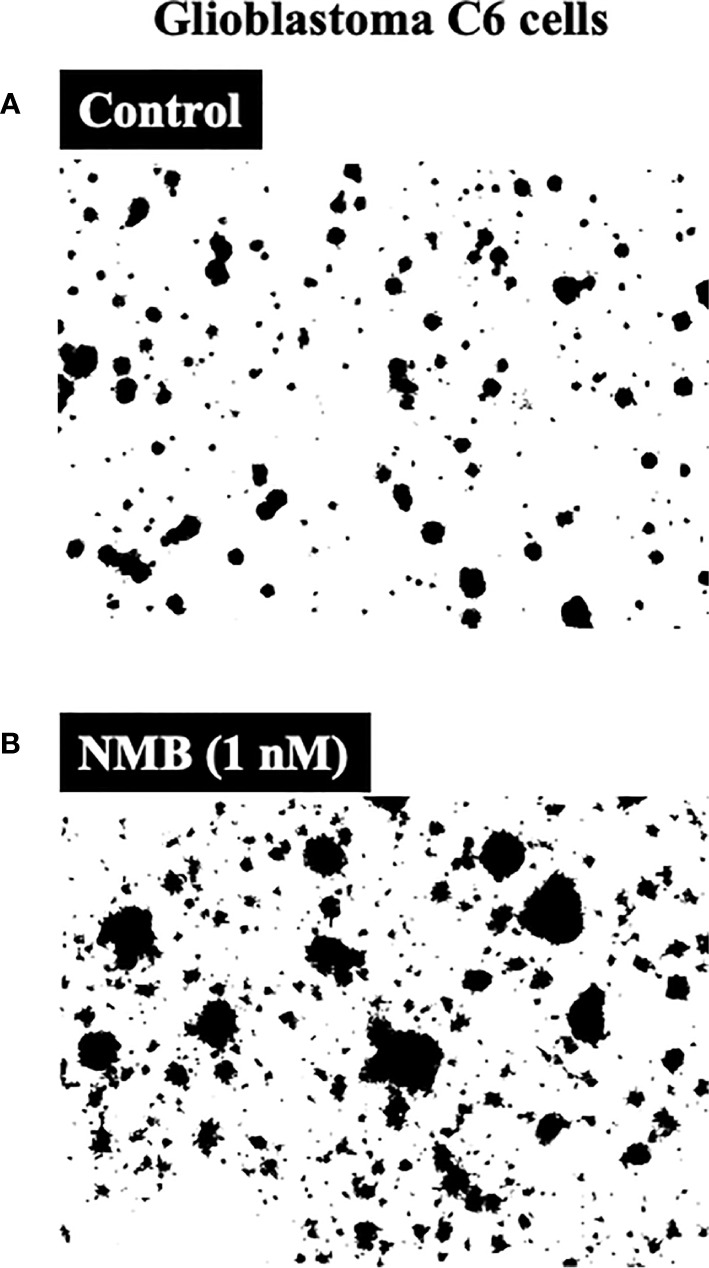
Ability of neuromedin B to stimulate growth of Glioblastoma C6-cells (bottom, panel **B**) compared to control (top, panel **A**). NMB (1 nM) stimulated a 177% increase (colony number increase from 74 ± 2 to 205 ± 9). Figure is drawn from data in (146).

In one study (146) the proliferation of rat C-6 glioma cells was stimulated by activation of the NMBR (both increased colonies in soft agar and increased cell numbers) (**Figure 2**), and the growth of these cells was inhibited by the selective NMBR antagonist PD168368 (45, 57, 154). However, in another study (149), the proliferation of the rat glioma C-6 cell line was stimulated by Bn (which can activate both GRPR and NMBR) (14, 39), and this effect was inhibited by the GRPR antagonist RC-3095. Preliminary data from a study of cultured human U-87 glioma cells (165) report BnR activation can lead to the development of brain tumor stem cell expansion with increased neurosphere formation, which may contribute to the increased presence of brain tumor stem cells in human gliomas.

#### Gliomas: Bn/BnR Related to Prognosis, Treatment

In studies with experimental gliomas, the treatment with GRPR antagonists increased the survival time by inhibiting the growth of xenografts of human glioblastomas cell lines (U-87G,U-373MG) in nude mice (159, 160).

In one study (138), the expression level of GRPR in gliomas (content, intensity, or area ratio) was not related to tumor grade, and the GRPR content index did not correlate with patient survival. Furthermore, data from the Cancer Genome Atlas Research Network, including mRNA from 206 human glioblastomas, found no difference in survival between patients with tumoral samples having high, compared to those with low, GRPR mRNA tumor levels (8, 166).

The overexpression of BnRs is increasingly being used to not only image tumor cells possessing these receptors but also to target cytotoxic compounds to the tumor cells (10, 11, 13, 15, 20, 133, 136, 137). Both of these subjects will be reviewed in a separate later paragraph, particularly covering recent studies in human CNS/neural tumors. A similar strategy has been studied experimentally in glioblastomas (167) by examining the effects of a cytotoxic Bn-analogue, AN-215 [i.e., a Bn carrier conjugated to the doxorubicin analogue, 2-pyrrolino-doxorubicin(AN-201)] on the human glioblastoma cell lines U-118MG, which expresses GRPR (145, 168), and U-87MG, which expresses both GRPR and NMBR (167) (**Table 1**). This cytotoxic analogue (167) bound with high affinity to U-87MG cells (IC_50_-4.1 nM), inhibited tumor growth by 70%, and increased the doubling time of the tumor almost twofold, whereas the unconjugated analogue AN-201 was ineffective. Similarly, treating human U-118MG glioblastoma cells with the cytotoxic Bn analogue AN-215 reduced tumor size by 50%, decreased tumoral VEGF levels by 45%, and decreased the relative ratio of Bcl-2 to Bax proteins by 90%, indicating a net apoptotic gain and showing the effectiveness of the therapy (168). A different approach (176) to BnR directed-targeted therapy involved using nanoparticles with anti-HIF-1-alpha siRNA targeted by incorporation of a Bn peptide. Systemic intravenous administration (176) of this BnR ligand to nude mice with U87 glioblastoma xenografts resulted in suppression of further tumor growth, silencing of HIF-1alpha expression by providing tumor specific siRNA tumor delivery (**Table 1**).

**Table 1:** Summary of studies using BnR over-/ectopic expression by gliomas/glioblastomas for targeting for tumor imaging (identification of primary/tumor extent) or for possible targeting with cytotoxic agents.

Tumor type	Purpose	BnR ligand used	Results	Author/year
**I. Human studies**				
A. Low-grade gliomas post resection, 9 pts	Imaging-?recur	^68^Ga-Bn/PET imaging with 18F-FDG uptake	Combination imaging identified in all, tumor recurrence/malignant change	Seiz et al. (169)
B. 7 pts with recurrent gliomas—uptake compared to tumor gene array analysis of tumor	Imaging	^68^Ga-BZH3 [DOTA-PEG_2_-(D-Tyr^6^,B-Ala^11^,Thi^13^,Nle14) BN(6– 14)] amide	All gliomas had uptake and correlated with tumor density of GRPR, not NMBR/BRS-3.	Strauss et al. (153)
C. Gliomas, 15 pts: 6 Grade II, 6 Gr III, 3 Gr IV	Imaging. Compare 18F-FDG	^68^Ga-BZH3 [DOTA-PEG_2_-(D-Tyr^6^,B-Ala^11^,Thi^13^,Nle14) BN(6– 14)] amide	^68^Ga Bn analog better than FDG/PET. Combo of FDG and ^68^Bn analog best discriminated low- from high-grade gliomas	Dimitrakopoulos-Strauss et al. (170)
D. 12 pts with gliomas/4=normals	Imaging	^68^GaNOTA-ACA-Bn(7-14)/PET imaging	All MRI lesions showed high ^68^Ga-Bn uptake. Correlation between tumor BnR expression and SUVmean uptake	Zhang et al. (171)
E. 8 children with suspected optic glioma	Imaging	^68^GaNOTA-ACA-Bn(7-14)/PET imaging	All 11 lesions (100%) in 8 pts seen with excellent contrast. Correlation of tumor BnR expression and SUVmean uptake	Zhang et al. (172)
F. Glioblastoma-26 pts	Tumor localization at surgery	IRDye800-Bn-fluorescent-labeled probed injected 16 h preop; detector used intraop	Probe: sensit=94%, specif=88% Conclude probe helped surgeon identify tumor border with high sensit/specificity	He et al./2021 (173)
G. 14 pts with glioblastomas	Tumor border localized at surgery	^68^Ga-IRDye800CW-Bn -near infrared fluorescent-labeled probe given preop detector used intraop	Excellent correlation between preop tumor location and probe signal at surgery. Probe: sensit=94%, specif=100%. Probe improved intraop tumor removal.	Li et al. (174)
**II. Animal studies**				
C6-glioma cells- orthotopic grafted rats	Nose-to-brain targeted delivery	Bn/PEG-Tat-conjugated micelles ± camptothecin (CPT)	Selective uptake seen Marked selective cytotoxicity Rats Tx with CPT micelles had greater survival	Kanazawa et al. (175)
Studied mice with orthotopic U87MG glioma xenografts	Tumor border localization at surgery	^68^Ga-IRDye800CW-Bn-near infrared fluorescent-labeled probe detector used intraop	Orthotopic tumors could be precisely removed using detection system	Li et al. (174)
Xenografts of U-118MG glioblastomas in mice	Targeted delivery for tumor cytotoxicity	AN-215[2-pyrrolino-DOX-14-O-glt-(13 Ψ14,CH2-NH, (Leu14]BN-(7-14)]	Reduced tumor growth by 50% Diminished VEGF by 45%, caused increased apoptosis.	Kanashiro et al. (145)
Xenografts of U-87MG glioblastomas in mice	Targeted delivery for tumor cytotoxicity	AN-215[2-pyrrolino-DOX-14-O-glt-(13 Ψ14,CH2-NH, Leu14)BN-(7-14)]	Reduced tumor growth by 70% Increased tumor doubling time to twofold longer	Szereday et al. (167)
Xenografts of U-87MG glioblastomas in mice	Targeted siRNA delivery for tumor cytotoxicity	Nanoparticles (ECHO-PEG-Bn analog-anti-HIF-alpha siRNA)	Prevented additional tumor growth Silenced HIF-1alpha expression and provided tumor specific siRNA tumor delivery	Wang et al. (176)

Bn, bombesin; Combo, combination of; Gr, tumor grade; FDG, ^18^F-deoxyglucose uptake; HIF-hypoxia inducible factor; Intraop, intraoperative; PET, positron emission tomography; preop, preoperative; Pts, patients; recur, question tumor recurrence; sensit, sensitivity; specif, specificity; Tx, treatment; VEGF, vascular endothelial growth factor.

The prognosis of patients with glioblastoma multiforme remains poor despite aggressive treatment with resection, or localized radiation with concomitant chemotherapy with temozolomide (27, 128, 166). A number of different prognostic gene panels from patient’s glioblastoma tumors have been found, and one of the most recent reported is a four-gene panel identified from analysis of the Cancer Genome Atlas-GBM database (177). This study involved the analysis of data from 686 patients with glioblastoma multiforme, with the initial identification of 133 different tumoral, differentially expressed genes associated with survival, which led to the final identification of a four-panel gene set. This four-panel gene set includes genes for neuromedin B (NMB), reticulon 1 (RTN1), glypican 5 (GPC5), and epithelial membrane protein 3 (EMB3) (177). Patients with glioblastoma multiforme with a low-risk tumoral four-gene panel had a better survival compared to those with a high-risk four-gene panel (p=0.035), and by both univariate and multivariate analyses, the four-gene panel result was an independent prognostic factor for survival in patients with glioblastoma multiforme (177).

### Bn-Related Peptides and Neuroblastomas

#### Neuroblastomas: General

Neuroblastoma is responsible for approximately 15% of all cancer deaths in children and is the most common extracranial solid tumor in children (95% <10 yrs. old) (28). Neuroblastomas arise from the sympathetic nerves or adrenal medulla with 70% occurring in the abdomen (178). Approximately 50% of patients have distant metastases at presentation, and although the disease course is highly variable, long-term survival in those with high-risk neuroblastoma (which is almost one-half) is approximately 40% overall and <20% in those with resistant or recurrent disease (178–180).

Neuroblastomas are frequently classified as neuroendocrine tumors (NET) because they can show neuroendocrine differentiation with immunocytochemical staining for NET markers (chromogranin, synaptophysin, etc.) (181, 182). In addition, similar to NETs, neuroblastomas can demonstrate amine precursor uptake and decarboxylation, as well as secrete numerous biologically active peptides/amine including vasoactive intestinal peptide, gastrin, catecholamines, serotonin, and GRP (181, 183–185).

#### Neuroblastomas: Bn/BnR Expression

Numerous studies report the presence of Bn-related peptides and BnRs in neuroblastoma tumor tissue and in neuroblastoma cell lines. In most, but not all, reports (186) that involve both PCR studies of GRP/GRPR mRNA expression (184, 187–190) and studies of immunoreactive GRP/Bn-related peptides (188, 191), they have reported their presence in neuroblastoma tumor tissue (188, 191) and in neuroblastoma cell lines [human IMR-32, SK-N-SH,BE(2)-C, murine Neuro2A cells] (100, 187, 188, 190, 192). In one study of 33 human neuroblastomas (188), 73% possessed GRPR by immunocytochemical studies, with increased expression in undifferentiated tumors compared to benign tumors. In this study of GRP (188), immunoreactivity was also detected in 73% of the neuroblastomas, but in contrast to the GRPR, its expression was not affected by the tumor histology. In another study (189), in tissues from 19 neuroblastomas, all had GRP mRNA and GRPR mRNA detected, although neither the amount of GRPR nor GRP mRNA correlated with prognosis. In studies of Bn immunoreactive cells in bone marrow aspirates from 36 patients with stage IV neuroblastoma, all of the patients had positive cells, with the degree of positivity varying from 72 to 96.2% of the cells (191), and there was a close correlation between results of the Bn immunoreactive assessment of the bone marrow aspirate and that of the primary tumor (193).

In seven human neuroblastoma tissues, using immunocytochemistry, GRP receptors were found (194). Using PCR, GRPR mRNA was found in three human neuroblastoma cell lines (IMR-32, SK-N-SH, LAN-1) (184). Similarly, in binding studies in human neuroblastoma cell lines, in a study using ^125^I-GRP combined with GRPR-crosslinking, SK-N-SH neuroblastoma cells were found to possess a GRPR of identical molecular weight as that on Swiss 3T3 fibroblasts (192).

#### Neuroblastomas: Bn/BnR Signaling

There are only limited studies of cellular signaling by activation of BnRs in neuroblastomas. GRP is reported to increase cytosolic calcium in neuroblastoma cell lines (SK-N-SH, LAN-2) (188), and in LAN-1 neuroblastoma cells, the stimulation was inhibited by a specific GRPR antagonist (188). In IMR-32 neuroblastoma cells, GRP did not stimulate changes in cytosolic calcium, although GRP stimulated growth, suggesting both Ca^2+^-dependent and -independent pathways may mediate the growth effects of GRPR activation in different neuroblastoma cells. Similarly, in the human neuroblastoma cell line LAN-2 (195, 196), stimulation with Bn resulted in activation of phospholipase C with the generation of inositol phosphates, which occurred by a pertussis toxin–sensitive G protein mechanism and was Ca^2+^ and PKC independent (195).

In the human neuroblastoma cell line BE(2)-C, Bn stimulated secretion of VEGF (197), which was mediated by PKC activation and not by the Bn-induced activation of the Akt (increased pAKT) or ERK (increased p-ERK1/2) signaling cascades, which was also seen with Bn treatment.

Two studies (184, 198) provide evidence for an important role in GRPR activation in neuroblastoma cell lines for the PI-3K pathway in mediating the GRP-stimulated growth effects in these cells. In human neuroblastoma cell lines LAN-1, SK-N-SH, and BE(2)-C, both GRP and Bn stimulate increased phosphorylation of AKT and GSK-3beta (198, 199), and these effects are inhibited by a GRPR antagonist or incubation with GRPR siRNA. In addition, incubation with the PI3K inhibitor LY294002 inhibited Bn-stimulated increases in pAKT and p-GSK-beta, as well as its cell cycle targets (198), Bn-stimulated increases in the G1/S cell cycle regulator cyclin D, and Bn-stimulated BrdU incorporation in these cells (198). In human neuroblastoma cell lines LAN-1, SK-N-SH, and BE(2) (198), GRP stimulation increases the expression of cyclin D and phospho-RB and decreases the expression of the cyclin-dependent kinase inhibitors p21 and p27 (198), which are consistent with GRP’s stimulated increases in the G1/S phase seen on flow cytometric studies and increases in DNA synthesis. The above studies support the conclusion that activation of the PI3K cascade plays an important role in neuroblastoma growth and cell survival. The tumor suppressor gene PTEN (phosphatase and tensin homolog deleted on chromosome 10) is a negative regulator of PI3K and plays an important role in growth and cell survival of various cancers (200). In 24 human neuroblastomas (201) examined by immunohistochemistry, a decreased level of PTEN protein expression was found in undifferentiated tumors compared to differentiated tumors, whereas the pAKT protein levels were similar in the two groups. In this study (201) with GRPR overexpressing in two human neuroblastoma cell lines (SH-N-SH and SH-SY5Y), there was decreased PTEN gene expression and increased expression of pAKT, suggesting that GRPR activation not only activated the PI3K signaling pathway but also downregulated the tumor suppressor gene PTEN, a negative regulator of PI3K (201). The importance of PI3K mediating GRPR-stimulated growth in these cells was supported by the finding that LY294002, a PI3K inhibitor, suppressed the increased growth seen in GRPR-overexpressing neuroblastoma cells, as did overexpression of PTEN in these cells (201, 202).

In cultured SK-N-SH and CE(2) neuroblastoma cells, Bn not only stimulated growth but also stimulated an upregulation of VEGF expression, demonstrating it was an important stimulator of the angiogenic pathway in these cells (100). In nude mice (100) with SK-N-SH or BE(2)-C neuroblastoma cell xenografts, Bn administration increased the growth of the neuroblastoma cell xenografts, increased VEGF expression, increased expression of PECAM-1 (a marker for microvessels), and increased p-AKT in the xenografts, which were all attenuated by the addition of a GRPR antagonist. Furthermore, in a neuroblastoma xenograft study (100), a GRPR antagonist decreased the plasma VEGF levels, which correlated with a decreasing tumor size (100). Furthermore, inhibition of PI-3K with wortmannin in neuroblastoma cells markedly decreased both GRPR mRNA levels and neurotensin receptor levels (184).

In the aggressive neuroblastoma cell line BE(2)-C, which possesses GRPR, the knockdown of GRPR resulted in a decrease in DNA synthesis, a cell cycle arrest at the G(2)/M phase, a decrease in cell proliferation, a change in cell morphology, and a downregulation of p-AKT, a crucial driver in cell survival and development of cell metastases (190, 199). In addition, the knockdown of GRPR in these cells decreased p-p70S6K and S6 key regulators of cell metabolism and increased expression of the tumor suppressor PTEN, a key inhibitor of the PI3K/AKT pathway (199).

Using a doxycycline inducible system to silence GRP in human neuroblastoma cell lines BE(2)-C and SH-SY-5Y cells, the autocrine effect of GRPR activation was investigated in these cells (202). Silencing of GRP generation/release resulted in decreased anchorage-independent growth, inhibition of cell migration and neuroblastoma cell-mediated angiogenesis, inhibition of the activation of the of PTEN/AKT signaling cascade, deceased mRNA level of various oncogenes (MYCN, TWIST, FAK), and suppression of the development of metastases (202).

In various neuroblastoma cell lines (SK-N-SH, LAN-1, IMR-32, BE(2)-C), evidence for the importance of other signaling cascades in mediating BnR-stimulated growth is reported. These include a study (194) reporting the importance of activation of focal adhesion kinase in mediating GRPR-stimulated growth of the human neuroblastoma cell lines BE(2)-C and SK-N-SH, as well Bn-stimulated formation of liver metastases *in vivo*. Second, in two studies (194, 203), GRPR activation in various neuroblastoma cells (SK-N-SH, IMR-32, LAN-1) stimulated the expression of matrix metalloproteinase-2 (MMP-2); a decrease in the expression of the tissue inhibitor of MMP-2; and the upregulation of integrin alpha 2, alpha 3, and integrin beta 1 proteins, as well as their mRNA expression; and a reduction of the integrin beta 1 inhibited GRPR-stimulated cell migration. In a third study (204), GRP silencing in the human neuroblastoma cell line BE(2)-C had a marked differential effect on the cell cycle regulators p21 and p27, with a 60% decrease in p21 and a threefold increase in p27. In addition, GRP silencing in these neuroblastoma cells (204) increased the expression and accumulation of PTEN in the cytoplasm, where it colocalized with p27, suggesting that p27 was functioning as a tumor suppressor by stabilizing PTEN in the cytoplasm. In a fourth study (205), GRP treatment of the human neuroblastoma cell lines SK-N-SH or BE(2)-C rapidly increased phosphorylation of the ET1 transcription factor, which correlated with increasing its transcription activity, resulting in an increase in Et1 nuclear accumulation and enhanced binding to its DNA consensus sequence. This in turn (205) resulted in increased expression and secretion of the proangiogenic factor interleukin-8 (IL-8), suggesting a critical role of this signaling cascade in GRP-induced angiogenesis in neuroblastomas and their development of metastasis (205).

#### Neuroblastomas: Bn/BnR Affect Tumor Growth/Proliferation

Numerous studies have reported effects on growth and proliferation or effects on cell viability of activation of BnRs on neuroblastoma cell lines (100, 187, 192, 198, 201–203). These studies involve both the use of selective GRPR antagonists (100, 187) and the effects of BnRs agonists, including both Bn (non-selective) or GRP (selective for GRPR) (100, 192, 198, 203). In almost all studies, selective GRPR antagonists inhibited cultured neuroblastoma cell line growth (198), as well as SK-N-SH xenograft growth in nude mice (100). However, in one study (187) involving the murine neuroblastoma cell line Neuro2A, a low concentration (0.1 nM) of the selective GRPR antagonist RC-3095 inhibited cell growth, but with a higher concentration (100 nM), it stimulated growth. In this study (187), the growth stimulatory effect of the GRPR selective antagonist was inhibited by HDAC inhibitors, suggesting it might be mediated by epigenetic mechanisms (187).

The GRPR selective agonist, GRP stimulated growth of human neuroblastoma cell lines [SH-SY5Y, SK-N-SH, IMR-32, LAN-1, BE(2)-C] in culture (100, 188, 192, 198, 201–203), as well as in xenografts (SK-B-SH cells) in nude mice (100, 202), and induced S-phase progression in SK-N-SH neuroblastoma cells (198). Overexpression of the GRPR in a human neuroblastoma cell line (SH-SY5Y cells) (201) markedly increased the basal growth rate. In IMR-32 neuroblastoma cells (188), GRP did not stimulate changes in cytosolic calcium, although it stimulated growth in these cells, suggesting both Ca^2+^-dependent and Ca^2+^-independent pathways may mediate the growth effects of GRPR activation in different neuroblastoma cells (188). Results in SK-N-SH neuroblastoma cells (188, 192) suggested that GRP can function in an autocrine growth manner in this neuroblastoma cell line, because conditioned media from these cells, when added to fresh SK-N-SH cells, stimulated significant growth, which was inhibited by the addition of an anti-GRP antibody (188), and the conditioned media contained high concentrations of GRP (192). This result is supported by an additional study (183), which demonstrated GRP content was high in conditioned media from malignant retroperitoneal tumors, including from a malignant neuroblastoma, which led the authors to conclude that GRP was likely functioning in an autocrine growth manner in these malignant tumors.

In neuroblastoma cell line BE(2)-C, in which activation of GRP stimulates growth (100), the knockdown of GRPR resulted in a decrease in anchorage-independent growth *in vitro* (199). In contrast, the overexpression of the GRPR in less aggressive SK-N-SH neuroblastoma cells resulted in colony formation, which was inhibited by GRP-blocking antibody (199). *In vivo*, xenografts in nude mice of BE(2)-C neuroblastoma cells, in which the GRPR was knocked down, showed delayed tumor growth, and these animals had markedly diminished liver metastases, providing evidence that GRPR in neuroblastoma cells had oncogenic properties, in addition to its mitogenic growth capabilities (199).

MYCN is amplified in 20–25% of neuroblastomas, and its amplification correlates with a poorer prognosis in these patients (28). Similarly, there is increased evidence that activation of the PI3K/Akt/mTOR signaling cascade (which occurs in 2/3 of cases) plays an important role in both the development and progression of neuroblastomas and is associated with a worse prognosis (206). There is increased evidence in neuroblastomas that these two signaling cascades (i.e., MYCN and PI3K) interact, which contributes to the poorer prognosis (206). In BE(2)-C neuroblastoma cells, knockdown of GRPR resulted in downregulated Akt2, which was associated with decreased cell proliferation (199). Furthermore, silencing GRPR in BE(2)-C neuroblastoma cells (190) reduced N-Myc expression by an Akt2-mediated mechanism. The silencing of AKT2 in cultured neuroblastoma cells resulted in decreased growth and decreased VEGF secretion *in vitro*, while Akt2-silenced xenografts in nude mice showed fewer liver metastases; therefore, Akt silencing caused the same results as knockout of GRPR in the neuroblastoma cells (190, 199). These results led the authors (190) to raise the possibility that targeting of the GRP/GRPR/Akt2 signaling cascade could be a novel therapeutic approach.

Some studies also support a role for NMB/NMBR in affecting neuroblastoma cell line survival, proliferation, and viability. In one study (207) similar to NGF, NMB administration prolonged the survival of the neuroblastoma cell line SH-5Y, although it did not promote neuron-like differentiation of these cells, whereas NGF did.

#### Neuroblastomas: Bn/BnR Related to Prognosis, Treatment

In one study (189), neither the expression level of GRP mRNA nor GRPR mRNA in the neuroblastoma tissue correlated with prognosis. However, in another study (188), the level of expression of GRPR mRNA, but not the amount of GRP mRNA, correlated with the histology of the neuroblastoma, with undifferentiated tumors having greater expression. This result was supported by a third study (183) of eight retroperitoneal tumors (including four neuroblastoma/ganglioneuromas, one Wilms tumor, one primitive neuroectodermal tumor, one rhabdoid, and one benign brachial plexus tumor) in which the GRP content in the conditioned media after incubation with each of the tumors was assessed, and it was found the GRP concentration was significantly greater (p=0.003) with all the malignant tumors (including the neuroblastoma) compared to the benign tumors. The authors (183) proposed that GRP could be a promising candidate tumor marker for malignant retroperitoneal tumors, including neuroblastomas. However, in a study (191) of predictive factors for relapse after treatment in 108 bone marrow preparations from 36 children with stage IV neuroblastoma, the degree of Bn immunoreactivity was not significant, whereas chromogranin A and NPY expressions were highly predictive of an unfavorable response.

In a 3-year-old child (208) with a malignant neuroblastoma with elevated levels of plasma GRP and pancreastatin, there was a 30–60% decrease in the high levels with treatment with octreotide, leading the authors to suggest that assessment of plasma GRP and other peptides might be useful markers of response to treatment.

The overall survival for high-risk patients with neuroblastoma remains <50%, despite aggressive treatment with radiation, surgery, chemotherapy, and immunotherapy (28), and thus there is a need for new approaches. Data from one experimental study (209) raise the possibility that silencing of GRP secreted by neuroblastomas, which can have an autocrine growth effect, could potentiate the use of chemotherapeutic agents. In this study (209) in two human neuroblastoma cell lines (JF, SSK-N-SH) in which GRP was silenced using siRNA targeting, there was a marked increase in neuroblastoma cell apoptosis and a decrease in cell proliferation. Furthermore, the combination of silencing GRP and chemotherapeutic agents (vinblastine, etoposide) resulted in enhanced apoptosis when compared to each alone, and led to the increased expression of proapoptotic proteins p53 and p21.

### Bn-Related Peptides and Medulloblastomas

#### Medulloblastomas: General

Medulloblastoma is the most frequent malignant brain tumor in childhood and accounts for 20% of all childhood CNS tumors (210–212). The incidence is approximately five per million in children and one per million in adults (211). Characteristically, medulloblastomas are highly aggressive tumors that occur in the cerebellum (210, 211). Medulloblastoma is now classified by WHO into four groups based on molecular profiling, which includes the following subtypes: Group I, the wingless (WNT) group; Group II, the sonic hedgehog group (SHH); Group III; and Group IV (29, 210, 211). The WNT group (Group I) has the best prognosis with a 90% survival rate and is the least common (10%), and 85–90% possess mutations in the *CTNNB1* gene that encodes for beta-catenin, which results in constitutive activation of the WNT pathway (210–212). Group II, the SHH subgroup, comprises 25–30% of all medulloblastoma cases and is most commonly is seen in children <3 or >16 years old. It primarily occurs in the cerebellar hemispheres and is due to SHH cascade mutations resulting in constitutive activation of this signaling cascade, and it has an intermediate prognosis (210–212). Group IIII accounts for 25% of medulloblastomas. The cell of origin is the neural stem cells; the elemental cause is not established but does not involve SHH/WNT aberrant activation. The most common mutations are in SMARCA4 and GABRA5, and it has high levels of MYC amplification. The 5-year survival is 39–58% (210, 211). Group IV is the most common, comprising 35% of cases. It originates from unipolar brush cells. No unique signature has been described, but a number of mutations are not infrequent (<20%) (KBTBD4, ZMYM3, KDM6A), with overexpression of PRDM6 and expression of the isochromosome 17q (80%), with an intermediate prognosis (29, 210–212).

#### Medulloblastomas: Bn/BnR Expression

In two studies (213, 214), three different medulloblastoma cell lines (DAOY, D283, ONS76) were examined for expression of GRPR by PCR and by immunohistochemistry. On immunohistochemical analysis, as well as PCR studies, all three medulloblastoma cell lines possessed GRPR and GRPR mRNA. A later study (213) of the medulloblastoma cell lines DAOY and D283 reported both cell lines expressed NMBR mRNA and NMB mRNA. In nine patients with medulloblastomas or with central primitive neuroectodermal tumors, four of the patients’ tumors (44%) had Bn-IR detected (205).

#### Medulloblastomas: Bn/BnR Signaling

No studies have examined the signaling cascades of BnRs (GRPR, NMBR) in medulloblastomas.

#### Medulloblastomas: Bn/BnR Affect Tumor Growth/Proliferation

Despite the presence by both PCR and immunohistochemical studies of mGRPR and GRPR, respectively, in the three different medulloblastoma cell lines (DAOY, D283, ONS76), treatment of the cells with either Bn or GRP did not affect cell viability (213, 214). In the human glioblastoma cell line U-138-MG, GRP stimulated proliferation only when combined with agents that increase cellular cAMP (forskolin, 8-Br-cAMP, PDE inhibitor), but proliferation was not seen when either agent was present alone (144). When a similar study was performed in the medulloblastoma cell line DAOY, the phosphodiesterase 4 inhibitor rolipram inhibited cell viability at all concentrations used (1–100 uM) and at a high concentration (100 uM) in the medulloblastoma cell line D283 (a Group III medulloblastoma) and ONS75 (214). Similar inhibition of viability was seen in DAOY cells when GRP was added to rolipram, as with rolipram alone, showing no growth effects of GRP in this medulloblastoma cell line (214).

In contrast to the lack of effect of activation of GRPR on growth of medulloblastoma cell lines (DAOY, D283, ONS76), the addition (213) of a NMBR antagonist (BIM-23127) (215) to the EGFR receptor antagonist cetuximab, at a concentration where cetuximab had no effect alone, reduced the viability of medulloblastoma DAOY cells. In contrast to the growth inhibitory effect of higher concentrations of cetuximab in the medulloblastoma cell line DAOY, neither the addition of NMB nor the NMBR antagonist BIM-23127 had an effect on the cell line’s growth or viability when present alone (215). These results suggested that NMBR activation alone does not affect DAOY medulloblastoma cell line’s viability, but instead could potentiate the growth inhibitory effect of EGFR blockade due to the addition of cetuximab (213).

Alterations in DNA methylation have been reported in medulloblastomas, similar to a number of other pediatric brain tumors (29, 216, 217), and there is increased interest in the use of histone deacetylase inhibitors [HDAC_i_] for their possible treatment (217–219). BnR antagonists in various tumor types have been shown to influence the effects of HDAC_i_’s, including having a potentiating effect on HDAC_i_-induced inhibition of growth of lung cancer cells (220), as well as a growth stimulatory effect in neuroblastoma cells, which is reversed by HDAC_i_s (187). In a study of the Group III medulloblastoma cell line D283 (218), the HDAC_i_ inhibitor, sodium butyrate reduced cell viability, whereas the NMBR antagonist BIM-23127 had no effect on cell viability alone, in contrast to the GRPR antagonist RC-3095, which stimulated growth of these cells (218). The combination of the HDAC_i_ and the NMBR or the GRPR antagonist gave similar results to the HDAC_i_ alone (187).

#### Medulloblastomas: Bn/BnR Related to Prognosis, Treatment

The principal treatment approaches in medulloblastoma (29) involve surgery, radiation, and adjuvant chemotherapy, most commonly with cisplatin, vincristine, cyclophosphamide, carboplatin, or lomustine.

There are no studies of Bn/BnR related to prognosis of patients with medulloblastomas. Similarly, there are no studies of Bn/BnRs in medulloblastoma patient’s treatment, only the limited studies related to growth or viability of medulloblastoma cell lines reviewed in the previous paragraph.

Similar to discussed in previous sections (i.e., *Introduction*/gliomas), in other CNS tumors (221–225), as well as medulloblastomas (213, 226–228), as in many other tumors (229), an important driver of proliferative/invasive/aggressive tumor behavior is the presence of EGFR mutations and amplification of various members of the EGFR receptor family (EGFR or ErbB1, HER-2 neu or Erb2, HER-3 or ErbB3, HER-4 or Erb4), particularly EGFR. In numerous cancers, therapies targeting various EGFR signaling cascade components are increasingly being used in EGFR expressing cancers (esp. lung, colorectal, etc.) (101, 103, 224, 229–234). As discussed in the previous sections, in other tumors, BnR activation can also play a major role in transactivation of member of the EGFR family (EGFR, HER-2, HER-3, HER-4) (101–103). The results of the studies reviewed above reporting the presence of GRPR/NMBRs on many medulloblastoma cell lines, coupled with the results of NMBR antagonism combined with EGFR blockade having potentiating effects reducing the viability of DAOY medulloblastoma cells (213), raise the possibility that the combination of BnR antagonists with other inhibitors of the EGFR cascade may be a novel approach worth exploring to control the growth of medulloblastomas in some patient subsets.

## Possible Role of BnR Ectopic/Overexpression by CNS/Neural Tumors for Tumor Imaging and for Treatment by Receptor-Mediated Therapy

### IV.A. BnR: CNS/Neural Tumor Imaging

#### BnR: CNS/Neural Tumor Imaging: General Comments

Tumor imaging is involved in all stages of the management of potentially malignant/malignant CNS/neural tumors (125, 235–239). Specifically, imaging is required at the initial stages of investigation to assess the location of the primary and extent of the disease, as this will determine the therapeutic approach (surgery, radiation, chemotherapeutic). If surgery is considered, it will be especially important to determine by imaging the extent of invasion to assess resectability; and if more extensive disease is present that is non-resectable, the location and extent of metastatic disease need to be carefully assessed prior to planning directed antitumor treatment and later to assess response to such treatment. Finally, post-treatment, periodic imaging studies are needed to assess recurrence and the extent of the recurrence.

While magnetic resonance imaging (MRI) remains the mainstay for initial and follow-up imaging of CNS/neural tumors and provides important information on tumor location, size, number of lesions, perifocal edema, and contrast enhancement, other aspects of tumor behavior are either not defined or incompletely defined, such as tumor heterogeneity, extent of metabolically active tissue, differentiating treatment-related changes from recurrent disease in previously treated patients, and extent of tumor invasion, in some cases (132, 239–242). Because of these latter limitations, there is increased interest in the development of other imaging approaches, particularly the use of positron emission tomographic (PET) techniques including [^18^F]Fluorodeoxyglucose (FDG) (132, 235, 239, 243), as well as other nuclear medicine approaches (132, 235, 243, 244).

#### BnR: CNS/Neural Tumor Imaging: Why Consider BnR-Based for These Tumors?

The use of G-protein-coupled receptors (GPCRs) is now receiving increasing attention (2, 10, 11, 13, 16, 135, 245) as a novel approach to both image various cancers and to deliver cytotoxic agents selective to the tumor (which will be discussed in a later section below for BnRs). This is occurring primarily because of the success of this approach in localizing neuroendocrine tumors using radiolabeled somatostatin ligands (2, 10, 242, 245). Neuroendocrine tumors overexpress somatostatin receptors (5, 246). With the development of ^68^Ga-labeled somatostatin analogues useful for PET imaging, somatostatin receptor imaging (SRI) has now become the most widely used imaging modality to image both the primary location of these tumors and the extent of the neuroendocrine tumor (2, 242), and has the highest sensitivity and specificity of any imaging modality for these tumors (10, 242, 247).

Unfortunately, somatostatin receptors are not ectopically expressed/overexpressed on the most frequent non-endocrine tumors, such as breast, prostate, pancreatic, etc., or CNS tumors (gliomas, medulloblastoma, etc.), except neuroblastomas, so that to use this imaging approach in these tumors, some other receptor family that is ectopically expressed/overexpressed needs to be used. A number of GPCRs are ectopically expressed/overexpressed by these tumors, but one of the most frequent is members of the BnR family, which are increasingly being studied for roles in imaging of a number of these tumors (particularly, prostate, lung, breast) (6, 8–11, 15, 22, 31, 89, 135, 142, 248). As discussed in the earlier sections of this paper, the BnRs are frequently ectopically expressed/overexpressed in a number of important CNS/neural tumors (gliomas, neuroblastomas, medulloblastomas), and thus there is increased interest in the use of Bn ectopic/overexpression for imaging these tumors (8–11, 15, 31, 135, 248, 249).

Although at this time there are no FDA-approved BnR imaging modalities, there have been many studies investigating the possible utility of various radiolabeled Bn agonists (11, 13, 16), and more recently antagonists (10, 11, 91), for cancer imaging. These studies involve a number of the frequent non-endocrine cancers, particularly the case of prostate cancer (10, 11, 15, 22, 73, 91), which ectopically express/overexpress GRPRs in 62–100% of cases in various series, breast cancer (10, 11, 15) (ectopically express/overexpress GRPRs in 40–75%) (9, 10, 14, 250), and as reviewed below, various CNS tumors, particularly gliomas.

#### BnR: CNS/Neural Tumor Imaging: Studies in Literature

A number of studies using different ligands for BnR receptor imaging have reported results in CNS/neural tumors, particularly in gliomas. A summary of the methods used, tumor preparations, and results for both imaging and targeted therapy in gliomas is listed in **Table 1**.

##### BnR: CNS/Neural Tumor Imaging: Studies in Literature: Glioma

There are two experimental *in vitro*/*in vivo* studies (174, 251) describing the interaction of novel BnR PET ligands with glioma cells. In one study (174), a PET/near-infrared fluorescence (NIRF) dual imaging modality GRPR ligand, ^68^Ga-IRDye800CW-BBN [the NIRF fluorophore IRDye800CW coupled to ^68^Ga-NOTA-ACA-BBN(7-14) (174)], for use in patients with gliomas both preoperatively and at surgery, was developed and first tested for its ability to image/localize U86MG glioma xenograft tumors in mice (174). In nude mice with brain U86MG glioblastoma brain xenografts, some remnant tumor tissue could not be detected by three experienced surgeons by the naked eye; however, after the injection of IRDye800CW and the assessment of the NIRF signal, the remnant tumor around the margins was easily identified and resected (174). In a second study (251), a three-dimensional (3-D) photoacoustic (PA) Bn ligand nanoprobe (NP), [(BBN-CuS)-NP], using the second near-infrared window (NIR-II) (1,000–1,700 nm) for detection, was described for localization of both a BnR-containing peripheral orthotopic tumor (prostate cancer cell line-C4-2) and for an orthotopic CNS glioma (C-6 glioblastoma cells). This probe utilizes a hybrid imaging approach that integrates optical excitation with ultrasound detection, allowing visualization of tissues at greater depths compared to traditional optical imaging methods (251). In this study (251), nanoparticles with Bn incorporated into the ligand enhanced the ability of the NIR-II PAs to image both orthotopic prostate cancer cells and intracranial orthotopic gliomas (C-6 glioblastoma cells) deep in both the prostate and the brain, respectively (251).

A number of recent studies in humans with gliomas have assessed the ability of various Bn analogs to image these tumors. In 12 patients with gliomas and 4 healthy volunteers (171), the safety, distribution, and radiation dosimetry were assessed after the administration of the radiolabeled BnR agonist ^68^Ga-NOTA-ACA-BBN(7-14). The administration of ^68^Ga-NOTA-ACA-BBN(7-14) was well tolerated, with no side-effects, the isotope was rapidly cleared primarily by renal excretion, and in all the glioma patients, the MRI-identified glioma showed a strong signal with the radiolabeled BN analogue ^68^Ga-NOTA-ACA-BBN(7-14) (171). The tumor-to-brain ratio was 24 ± 8.8 based on the SUV_max_, and immunohistochemical analysis of GRPR tumor expression showed a positive correlation with its intensity with the radioisotope’s *in vivo* SUV (r=0.71, p<0.001) (171). In a separate study (172) in eight children with suspected optic gliomas, the ability of the same radiolabeled Bn analogue used in the study above, to localize a possible tumor, was assessed. Eleven lesions were well localized in the eight patients, and the mean tumor-to-brain SUV_max_ on PET scanning was higher with the ^68^Ga-NOTA-ACA-BBN(7-14) than with ^18^F-FDG PET (28.4 ± 5.6 *vs* 18.2 ± 5.0) (172). All lesions were pathologically confirmed, all had GRPR expression, 75% were pilocytic astrocytomas-Grade I, and 25% were diffuse astrocytomas-Grade II (172). Similar to the previous study, there was a positive significant correlation between ^68^Ga-NOTA-ACA-BBN(7-14) SUV_mean_ and the expression level of GRPR in the gliomas (r=0.56, p<0.05) (172).

Recent studies (153, 169, 170) have reported promising imaging results in patients with gliomas using the high-affinity radiolabeled GRPR PET ligand ^68^BZH3 [^68^Ga -DOTA-PEG2-[DTyr^6^,betaAla^11^,Thi^13^,Nle^14^]Bn(6-14)] (252). In one study (153) involving seven patients with recurrent gliomas, the kinetics and imaging results of tumor identification of ^68^BZH3 were studied, and the results correlated with the expression of BnRs in the tumors (GRPR, NMBR, BRS-3), assessed by gene array studies. ^68^BZH3 accumulation in all the gliomas could be determined, and the presence of all three BnR subtypes could be found in all the gliomas by the gene array studies (153). In the kinetic analysis of this study (153), the rate constant for global receptor binding, K_1_, correlated (r=0.89, p<0.05) directly with the gene array expression data for GRPR, but not for NMBR or BRS-3. In a second study (170), the pharmacokinetic parameters as well as the imaging results with ^68^BZH3 and ^18^F-FDG PET were compared in 15 patients with histologically confirmed recurrent gliomas. This study included six patients with WHO II gliomas, six with WHO III gliomas, and three patients with WHO IV recurrent gliomas (170). Ten (67%) of the 15 patients demonstrated positive ^68^BZH3 PET scans for glioma, and 6/15 (40%) a positive ^18^F-FDG PET scan. The median SUV for ^18^F-FDG PET was higher in both the low- and high-grade tumors than that for ^68^BZH3 PET. The median SUV for ^18^F-FDG PET did not different between the low/high grades, whereas with ^68^BZH3 PET, the SUV was higher in the high-grade tumors than the low-grade tumors (170). A discriminant analysis found that the combination of the FDG influx and binding potential of ^68^BZH3, best distinguished between low-grade gliomas and those with high grade, with a correct classification in 100% (170). This finding may have particular significance, because in patients with low-grade or recurrent gliomas, frequently, neither MRI nor ^18^F-FDG PET can adequately distinguish glioma grade (170), and thus the addition of ^68^BZH3 may help in this important clinical separation of these two groups of gliomas. A third study (169) examined whether the use of ^68^BZH3 PET imaging could better differentiate, when compared to ^18^F-FDG PET scanning, the malignant transformation of a glioma from tumor recurrence in nine consecutive patients with low-grade gliomas, after surgical treatment and postoperative serial MRI imaging identified possible new lesions. In all cases showing proven malignant transformation, there was increased uptake on the ^68^BZH3 PET scan, whereas the ^18^F-FDG PET scan showed either a decrease or only a small increase (169). Furthermore, in the cases with no transformation and instead a recurrence only, the ^68^BZH3 PET scan showed no increase or even a decrease in uptake; therefore, only the ^68^BZH3 PET scan discriminated between these two important tumor growth courses, which have different treatment/prognostic implications.

Two studies have used a different approach to visualizing BnRs on gliomas by forming fluorescence BnR-conjugated PET ligands, which can be used preoperatively or at surgery (173, 174). The BnR ligand ^68^Ga-IRDye800CW-BBN is a PET/near-infrared fluorescence (NIRF) dual-imaging modality GRPR ligand developed to fulfil these two requirements. After establishing that this NIRF ligand precisely localized U86MG glioma xenograft tumors in mice (174), it then was used in 14 patients with glioblastoma multiforme (174). Particular attention was paid to the potential value of this BnR ligand for preoperative tumor location and its ability to localize tumor margin at the time of surgery, because complete resection of glioblastomas is associated with improved survival but can be difficult to accomplish at surgery, because of their aggressive, infiltrative growth behaviors make it difficult to determine accurately the tumor margins and fully resect the tumor (173, 174). ^68^Ga-IRDye800CW-BBN preoperative PET scanning and intraoperative NIRF signals were evaluated, and an excellent correlation between these two methods for tumor localization was found (174). In both the experimental preclinical study in nude mice with glioblastoma xenografts and in the human studies, the fluorescence signals were higher in the gliomas than in the adjacent brain tissue (174). When the pathology results were compared in 43 loci to the NIFR-guided sampling results, the sensitivity was 94%, specificity 100%, and there were no side-effects with the ^68^Ga-IRDye800CW-BBN administration. The PFS at 6 months was 80% with two newly diagnosed glioma patients achieving long-term survival (174). In a second study (173), the ability of the near-infrared fluorescence (NIRF) GRPR ligand IRDye800CW-BBN to localize glioblastoma multiforme tumors at surgery in 29 patients was evaluated. The IRDye800CW-BBN was administered prior to surgery, the NIRF signal assessed at the time of surgery, and the surgical result assessed by postoperative MRI scanning (173). Complete resection of the glioblastomas was achieved in 83% of the patients, and the sensitivity of NIFR in the pathologic section for detection of the glioblastoma tumors was 94% and the specificity was 88% (173). This approach resulted in an overall survival and PFS rates of 23 and 14 months, respectively. The authors concluded that the use of this fluorescence probe at surgery assisted the neurosurgeon in identifying the tumor boundaries, and the results suggested its routine use may improve survival outcomes (173).

##### BnR: CNS/Neural Tumor Imaging: Studies in Literature: Medulloblastoma/Neuroblastoma

There are no studies published that have assessed the potential value in imaging by using BnR overexpression in either medulloblastomas or neuroblastomas, which occurs frequently as reviewed earlier in this chapter. That overexpression of the GPCR BnR may be effective in these CNS/neural tumors, as it has been in gliomas, is supported by recent studies with neuroblastomas. Neuroblastomas are classically imaged with MRI scanning, and to determine tumor extent/metastases, SPECT/CT scanning with meta[^123^I]iodobenzylguanidine ([^123^I]MIBG) is used (235). [^123^I]MIBG scanning is successful, because MIBG is a norepinephrine analogue, which is taken up by the sympathico-medullary tissue, from which these tumors are derived, which possess the norepinephrine transporter (235). However, approximately 10% of neuroblastomas are negative with [^123^I]MIBG SPECT/CT scanning, and the resolution with [^123^I]MIBG SPECT/CT scanning is relatively low, resulting in limited sensitivity for smaller lesions (235). Recently, these neuroendocrine tumors have been found to overexpress somatostatin receptors (sst2, primarily), which are GPCRs, similar to BnR, and that using this overexpression to image these tumors gives comparable or even better imaging results than seen on [^123^I]MIBG PET scanning, because the PET/CT scanning after ^68^Ga radiolabeled somatostatin analogues gives superior resolution (235, 244).

### BnR: CNS/Neural Tumor Treatment by Receptor-Mediated Therapy Imaging

#### BnR: CNS/Neural Tumor: Receptor-Mediated Therapy Using Radiolabeled BnR Ligands

The are no studies on BnR-mediated RMT using radiolabeled BnR ligands in gliomas, medulloblastomas, or neuroblastomas. However, a number of points suggest that it is very likely this will change in the near future and will become an increasingly studied area in patients with advanced disease from these tumors. First, the prototypical overexpressed GPCR that is now widely clinically used for antitumor treatment is the somatostatin receptor (primarily sst2 subtype), for the treatment of malignant neuroendocrine tumors (NETs), using radiolabeled (primarily ^177^Lu-, and to a lesser extent ^90^Y) somatostatin receptor ligands for Peptide Radio-Receptor Therapy(PRRT), in patients with advanced disease (253–263). The FDA approval of this was based on results of a double-blinded, control phase 3 trial (NETTER-1) (264) in patients with advanced unresectable, midgut carcinoid NETs, which demonstrated with PRRT treatment with a ^177^Lu-labeled somatostatin agonist analogue, PFS was significantly prolonged (from 8.4 to >40 months, p<0.0001), with an increased overall survival from 3 to 18%, combined with the results of treatment of 510 patients with advanced panNETs and NETs in other locations, treated in Rotterdam (265). In the latter group of patients, PRRT resulted in a complete response in 2%, partial response in 28%, and tumor stabilization in 35% (265, 266). In a recent meta-analysis of 22 PRRT studies in patients (1,758 patients) with various advanced NETs treated with PRRT, the pooled disease response rate (complete/partial tumor response) was 33% with RECIST criteria, and the pooled disease control rate (compete/partial response or stable disease) was 79% (267). As result of these studies in patients with advanced NETs, PRRT has become one of the main antitumor approaches now widely used in patients with advanced neuroendocrine disease (253–263). Second, with the PRRT success with radiolabeled somatostatin-analogues in treating malignant neuroendocrine tumors, this approach is being either applied or increasingly considered for use in other overexpressing somatostatin receptor–expressing malignant tumors (4, 10, 74, 235). However, because of the more limited distribution of somatostatin receptors in primarily endocrine-related tumors, there is rapidly increasing interest in using PRRT with other GPCR receptors frequently expressed in non-endocrine malignant tumors, such as BnR (10, 17, 20, 42). There are now numerous studies examining the cytotoxicity/efficacy of radiolabeled BnR analogs (primarily with ^177^Lu) in prostatic cancer, both in *in vitro* studies, as well as in animal studies and in patients with advanced prostatic cancer (10, 20, 73, 268), which shows promise for this approach. There are also numerous studies of PRRT with radiolabeled BnR analogues in patients with breast cancer (17, 23, 25, 73, 269), and other non-endocrine cancer cells (73), which show promise. Third, because of the success of tumor localization and PRRT-mediated cytotoxicity with radiolabeled somatostatin analogues in NETs, as well as limited studies showing promise with PRRT with radiolabeled somatostatin analogues in some patients with symptomatic/malignant CNS/neural tumors (meningiomas) (270–273), malignant neuroblastomas (274, 275), there is increased interest in applying nuclear medicine approaches to other CNS/neural tumors (125, 235, 240, 276, 277).

#### BnR: CNS/Neural Tumor: Receptor-Mediated Therapy Using Non-Radiolabeled Cytotoxic BnR Ligands

Because of the success of PRRT in NETs using radiolabeled somatostatin receptor ligands for administering targeted cytotoxicity to the tumor (reviewed in the previous paragraph), coupled with the lack of overexpressed somatostatin receptors in most common tumors, there is a marked increase in the possibility of also developing this therapeutic approach with other GPCRs in which the active ligand contains cytotoxic non-radioactive moieties that would result in receptor-mediated tumor cytotoxicity (RMT) on various malignant tumor cells (4, 10, 42, 74, 90, 262, 278).

Because BnRs are one of the most frequently overexpressed GPCRs on the more common malignant tumors (8–10, 13–18), numerous non-radiolabeled BnR ligands have been described to demonstrate cytotoxicity for various tumors. These non-radiolabeled cytotoxic BnR ligands are almost entirely directed at tumor overexpression of GRPRs and are composed of both GRPR agonists and antagonists (13, 14). Furthermore, the non-radiolabeled BnR tumoral cytotoxic analogs described have utilized a wide range of cytotoxic agents (10, 11, 90), including coupling to established chemotherapeutic agents (doxorubicin, paclitaxel/other taxol analogs, camptothecin, epigallocatechin) (23, 24, 79, 167, 168, 278–280) (**Table 1**); various marine toxins (hemiasterlin, dolastatin) (281, 282); to various photosensitizing/photothermal agents for administration of cytotoxic photodynamic therapy (10, 283); to various cell-penetrating cytotoxic agents (74, 175, 284); to the antimicrobial peptide magainin (285); to various siRNA constructs (10, 176, 286, 287); to various mitochondrial disruptive agents (10, 13); and to various cytotoxins such as diphtheria toxin (10, 13, 288). Recently, an effective cytotoxic agent *in vitro* and *in vivo* in rats with intracranial orthotropic C6-glioblastoma tumors (175) was described, which was made by conforming micelles containing the cell-penetrating agent Tat, which has been shown to enhance treatment of brain tumors in experimental studies (249), with a BnR agonist peptide. Specifically, in this study (175), utilizing a nose-to-brain delivery approach *in vivo*, poly(ethylene glycol)-polycaprolactone-blocked micelle carriers were used, which were modified by addition of a cell-penetrating peptide, Tat (PEG-PCL-tat), which had been shown to have improved efficacy for the delivery of the chemotherapeutic agent camptothecin in the brain glioblastoma tumor model. To enhance selectivity for the glioblastoma, mixed micelles were prepared by combining the PEG-PCL-tat micelles with stearoyl-modified bombesin (Bom/PEG-PCL-Tat) (175), and the mixed micelles had enhanced selectivity, cellular uptake, and cytotoxicity in isolated C6-glioblastoma cells. Furthermore, *in vivo* administration of Bom/PEG-PCL-Tat (175) resulted in marked tumor cytotoxicity with selective uptake by the brain glioblastomas, which resulted in enhanced survival of the treated intracranial orthotropic tumor-bearing rats.

## Discussion/Conclusions

The studies reviewed above demonstrate in some CNS/neural tumors [Gliomas (100%), medulloblastomas (45%), neuroblastomas (75%)], the BnR family of receptors are ectopically expressed/overexpressed. The converse of this statement should not be assumed, which is the BnRs were not found in other brain tumors. Unfortunately, this area has not been well-studied, and there are no systematic studies examining their occurrence/frequency in a large panel of different brain tumors. Most studies support the conclusion that GRPR (BB2) is the predominant BnR present on these tumors, and although evidence for NMBR (BB1) on some glioblastoma cells also exists, this also has not been systematically studied. In only one, small study (153) was the expression of all BnRs in a group of CNS/neural tumors examined. In that study (163), the mRNA expression levels were determined of each of the human BnRs (GRPR, NMBR, BRS-3) in recurrent gliomas in nine patients. In all patients, all three BnR subtypes’ mRNAs were found in all tumors, with the highest levels for BRS-3 (BB3), followed by GRPR, and the lowest for NMBR (153). The relative expression levels of the different BnRs in the different gliomas did not correlate with each other and thus were independently expressed for each of the three genes (153). Unfortunately, there are no other studies involving CNS/neural tumor BRS-3 receptors, which corroborate these findings.

In studies of both gliomas (human cell lines, rat C6-glioma cells) and human neuroblastoma cell lines (i.e., there were no studies of BnR signaling in medulloblastomas), BnR agonists stimulated activation of numerous cellular signaling cascades. In both gliomas and neuroblastomas, the signaling cascades activated were similar to results from detailed studies of activation of BnRs (GRPR/NMBR) in other cells (9, 12, 14, 19, 40, 47, 71, 96–100). In human gliomas, the primary signaling response is activation of the phospholipase C cascade, as well as MAPK activation, which was primarily mediated by activation of GRPRs. In rat C6-glioma cells, the predominant BnR present was NMBRs, whose activation also resulted in activation of phospholipases C/D, MAPK, and stimulation of tyrosine kinase kinases such as activation of p125FAK. Detailed stoichiometric studies (150, 151) of the ability of NMB to occupy the NMBR and stimulate cellular signaling cascades on C6-glioma cells compared to coupling of human NMBR in normal cells (**Figure 1**) demonstrated identical coupling for all the signaling cascades. Similarly in these studies, the NMBR in C6-glioma cells underwent agonist-induced internalization, downregulation, and desensitization in a similar manner to NMBRs on normal cells (12, 96–98, 110, 111, 151). In human neuroblastoma cell lines, BnR agonists (*via* GRPR) stimulated phospholipase C activation (188, 195, 196), which was by a pertussis toxin-sensitive G protein mechanism and was Ca^2+^ and PKC independent (195). Furthermore, stimulation of GRPRs on various neuroblastoma cell lines activated the PI3K/Akt pathway, which played an important role in mediating GRPR-stimulated growth (198, 199).

In gliomas (**Figure 2**) and neuroblastomas, numerous studies report that activation of BnRs has a prominent effect on tumor growth, DNA synthesis, migration, stimulation of angiogenesis, aggressiveness, and the development of metastases (194, 202). In contrast, there is very limited data on whether BnR activation in medulloblastomas has a growth effect. In gliomas and neuroblastomas, the above conclusions are supported by both use of selective BnR antagonists and by methods of silencing the BnR(siRNA), as well results from studies using selective agonists. There are numerous classes of selective GRPR/NMBR antagonists (12, 14, 30, 31, 44, 47, 48, 53, 54, 56, 57, 93), and in human glioblastoma cell lines, various GRPR selective antagonists were the most effective at inhibiting growth (138, 143, 145, 149, 159, 160, 162). In contrast, in some studies (149, 161), with rat C6-glioblastoma cells, a GRPR selective antagonist inhibited the growth, whereas in other studies, the growth was inhibited by NMBR antagonists (45, 57, 154). In human neuroblastoma cell lines or *in vivo* studies with primarily xenografts, numerous studies (100, 184, 197, 198, 201, 202) support the conclusion that activation of GRPRs, not NMBRs, is mediating the growth effects, as well as stimulating the increased migration and metastatic potential of the neuroblastoma cells.

The BnR-stimulated signaling cascades mediating growth in both neuroblastomas and gliomas were generally similar to the growth cascades described in non-CNS tumors and normal tissues (9, 10, 12, 16, 99, 101, 102). Specifically, in gliomas, stimulation of BnRs mediated growth by activation of the signaling cascades, which occurred primarily by stimulating VEGF secretion, angiogenesis, altering cellular oncogene expression/activity (c-fos, c-jun), altering cellular apoptotic status, effecting p53, p21, and p16 expression, as well as EGFR activation (145, 154, 160, 162, 164, 168) and brain stem cell expansion (165). In human neuroblastoma cell lines, activation of GRPRs had important growth effects by activation of VEGF expression and secretion; increased expression of PECAM-1 (a marker for microvessels); stimulating cell cycle G(2)M phase, p-p70S6K, and S6 (key regulators of cell metabolism), expression of oncogenes, expression of cell cycle regulators (p21, p27), and phosphorylation of the ET1 transcription factor, which correlated with increasing its transcription activity (100, 190, 197, 199, 204, 205).

Two important areas involved in BnR activation, that have implications for treatment in CNS/neural tumors, remain unclear and largely unstudied, which have been well-studied in BnR activation and growth stimulation in non-CNS neural tumors (14, 19, 71, 101–103). These two unresolved areas include whether the growth effects of BnR activation in gliomas/neuroblastomas are mediated by an autocrine growth mechanism, and second, whether BnR transactivation of the EGFR/HER receptor family plays a role in BnR’s growth stimulatory effects in these cells. In many, different non-CNS/neural tumors, the tumor growth stimulatory activity of the BnR occurs by an autocrine mechanism, wherein the tumor synthesizes and releases GRP/NMB-related peptides, which then activate the BnRs on the same cells (8–10, 12, 87, 88). In most cases, the autocrine mechanism is particularly important in the BnR activation and growth stimulatory effects (8–10, 12, 87, 88).

The results of studies evaluating an autocrine role of BnR activation in gliomas and neuroblastomas are limited and conflicting, whereas there are no data in medulloblastomas. In gliomas, one study in U-87MG glioma cells (160) reported both GRPR and NMBR mRNA were present; however, GRP mRNA was not found, leading the authors to suggest that the GRP effect on growth of this glioma cell line was likely mediated by a paracrine mechanism. This result is somewhat surprising, both because autocrine growth, a mechanism of activation, is present in so many other tumors and also because in gliomas, autocrine mechanisms are reported for a number of other glioma growth factors, including the G-protein coupled receptor CCR5 [with its ligand CCL5 (RANTES) present in glioma cells] (289), TGFbeta1 (290), bone morphogenetic proteins (BMP4) (291), PDGF (292), and dopamine (293). In contrast, in neuroblastomas, numerous studies support an autocrine role for Bn-related peptides in BnR activation. In studies (202, 209) silencing GRP generation in neuroblastoma cells, this resulted in decreased tumor growth, migration, angiogenesis, as well as a suppression of the development of metastases. In SK-N-SH neuroblastoma cells (184, 188, 192), GRP functions in an autocrine growth manner, because conditioned cell media, when added to fresh SK-N-SH cells, stimulated growth, which was inhibited by an anti-GRP antibody (188), and the conditioned media contained high concentrations of GRP (192). This result is further supported by an additional study (183), which demonstrated GRP content was high in conditioned media from malignant retroperitoneal tumors including neuroblastomas, which led the authors to conclude that GRP was likely functioning in an autocrine growth manner in these malignant tumors.

Alterations of the EGFR family of receptor tyrosine kinases (RTKs) are frequent in glioblastomas (222, 223), occurring the most frequently of any RTK (4× rate of PDGFRalpha) with 57% of all glioblastomas showing evidence of an EGFR mutation, rearrangement, altered slicing, and/or amplifications (222, 223). Of the four subtypes of glioblastomas, EGFR amplification/mutation is particularly associated with the classical subtype (222, 223). Deletions in the EGFR also occur frequently in glioblastomas with some deletion mutants being oncogenic (223). Deletion of EGFRvIII (lacking amino acid residues 6–273) occurs most frequently in glioblastomas, lacks a ligand binding domain, but is constitutively active (222, 223). In addition, point mutations occur frequently in the extracellular domains, which are activating (223).

The unclear role that BnR transactivation of the EGFR/HER receptor family plays in BnR’s growth stimulatory effects in gliomas or neuroblastomas is entirely due to a lack of studies. Numerous studies have documented an increasing role of BnR transactivation of the EGFR/HER family (EGFR/HER2/HER3/HER4) in various non-CNS/neural tumor cell growth, migration, invasion, resistance to drug therapy, and development of metastases (14, 71, 101–103). Only one study, in human glioblastoma cells (human A172 cells) (162), explored the ability of BnR stimulation to alter EGFR activation, which reported GRPR silencing by knockdown with a short hairpin interfering RNA sequence in these cells resulted in decreased cell growth associated with activation of EGFR, as well as an increase in p53, p21, and p16 cell cycle regulators; changes the authors proposed were consistent with GRPR silencing inducing cell senescence. These results are the opposite of what is generally reported in growth of other non-glial tumor cells stimulated by a BnR-dependent mechanism, where GRPR stimulation is found to stimulate, and GRPR inhibition to inhibit, activation of EGFRs (14, 71, 101–103). These results also differ from results of other studies on the mechanisms of growth stimulation of various glioma cell lines with activation of other GPCRs (cannabinoid, PGE2, formyl peptide receptor agonist) (294–296) or other growth stimulants such as phorbol esters/HSP90alpha (297, 298), which were found to be mediated by transactivation of the EGFR and stimulation of the EGFR signaling cascade in these cells (294–298). Additional studies are needed to resolve the exact role of BnR transactivation of EGFR/HER members in mediating glioblastoma growth/aggressive behavior.

Expression/activation of the EGFR/HER family has important effects on the growth/differentiation/aggressiveness of neuroblastomas. EGFR/HER1 was found in 100% of 13 neuroblastoma cell lines with its activation resulting in cell proliferation, with more cells entering S and G2-M phases (299). Furthermore, in high-grade neuroblastomas, increased activity of EGFR is one of the molecular changes associated with increased metastatic potential and poor prognosis (300). In neuroblastomas, there are no studies addressing the issue of whether the ectopically expressed/overexpressed BnR’s activation transactivates the EGFR signaling cascades in these cells to stimulate growth/migration or aggressiveness. From the available data from other growth stimulants’ mechanisms of action on neuroblastoma cells, as well as from studies on other NETs, which neuroblastomas are classified as, one would predict that it will likely be found that activation of BnRs in these cells will transactivate the EGFR/her family, and this will be involved in mediating growth effects in these cells. In various gastrointestinal NETs, which share many features with neuroblastomas, the activation of BnRs, as well as the activation of a number of other GPCRs, stimulates growth by transactivating EGFRs (104). Furthermore, transactivation of the EGFR/HER family has been reported as a frequently used mechanism by a number of different neuroblastoma growth stimulants, including activation of various GPCRs (muscarinic cholinergic receptors, prostaglandin E2 receptor EP4, dopamine D_2_ receptor), and by the urokinase receptor (301–304). Similar to the situation with gliomas, additional studies are clearly needed to resolve the exact role of possible BnR-transactivation of EGFR members in mediating neuroblastoma growth/aggressive behavior.

The ectopic expression/overexpression of BnRs (principally GRPR) in most gliomas and neuroblastomas (lesser extent medulloblastomas), combined with the marked effects of activation of these receptors on the tumor’s behavior (growth, migration, invasiveness, development of metastases), opens numerous novel possibilities for the treatment of these aggressive tumors. At present, these possibilities are largely underinvestigated. These possibilities are important to investigate in these tumors because these tumors are not only some of the most common CNS/neural malignant tumors, but they are also some of the deadliest, with a 5-year survival rate for glioblastomas of only 5% (26, 27, 127, 128). Similarly, neuroblastomas are the most common extracranial solid tumor in children (28) and are responsible for 15% of all cancer deaths in children (178–180). Therefore new, novel treatment approaches are needed with both of these tumors (27, 28, 142, 235, 243). BnR-based treatment possibilities in these CNS/neural tumors could be considered in four general areas. First, because of the profound antigrowth effects of neutralization of BnR activation in experimental *in vitro* and *in vivo* studies of these tumors, such an approach inhibiting BnR activation either at the receptor level or key signaling intermediates should be explored, either alone or in combination with other treatment approaches such as chemotherapy. Second, the BnR ectopic expression/overexpression by these tumors can be used for PRRT using cytotoxic radiolabeled Bn compounds, in a similar manner to the use of radiolabeled somatostatin receptor ligands, which are currently now widely used in the treatment of malignant neuroendocrine tumors over-/ectopically expressing somatostatin receptors, and is being increasingly examined in other non-endocrine tumors (prostate, breast, etc.) (2, 10, 11, 13, 16, 20, 92, 102, 135, 245, 262). In studies on non-CNS tumors, PRRT has been successful both when used alone (4, 11, 20, 21, 42, 73, 74) and in combination with other cytotoxic therapies, including chemotherapy (284, 305–309). Third, the BnR ectopic expression/overexpression by these tumors can be used also for targeted delivery of cytotoxic non-radiolabeled compounds including chemotherapeutic agents, which is increasingly being studied in other malignant tumors (4, 10, 13, 42, 74, 78, 79). Fourth, studies with NETs using ectopic expression/overexpression of somatostatin receptors to image the tumor is now the most sensitive method to localize these tumors (242, 246, 262). Using a similar strategy with BnR ectopic expression/overexpression by these CNS/neural tumors may allow better definition of infiltrative tumor margins at surgery (173, 174), which can be a major problem with gliomas, as well as allowing better definition of the extent of remaining tumor, which can affect the therapeutic approach.

In summary, the studies reviewed in this paper support the conclusion that some CNS/neural tumors (gliomas, neuroblastomas, medulloblastomas) have the high rate of occurrence of over-/ectopic expression of the BnR family, and their activation can have prominent effects on tumor growth behavior. These findings open the possibility of novel therapeutic approaches using this receptor family’s ectopic expression/overexpression. Particularly, the possibility of using BnRs’ presence for imaging and various forms of targeted therapy, which has been used so successfully with somatostatin receptors in malignant neuroendocrine tumors, is now being increasingly investigated with promising results, with other overexpressed GPCRs in other tumors, including BnRs.

## Author Contributions

All authors listed have made a substantial, direct, and intellectual contribution to the work and approved it for publication.

## Funding

This research was partially supported by intramural funds from the NIDDK and NCI, National Institutes of Health.

## Conflict of Interest

The authors declare that the research was conducted in the absence of any commercial or financial relationships that could be construed as a potential conflict of interest.

## Publisher’s Note

All claims expressed in this article are solely those of the authors and do not necessarily represent those of their affiliated organizations, or those of the publisher, the editors and the reviewers. Any product that may be evaluated in this article, or claim that may be made by its manufacturer, is not guaranteed or endorsed by the publisher.
